# Training on the care of patients with respiratory syndrome of middle east-coronavirus and ebola virus based on clinical simulation

**DOI:** 10.1186/2197-425X-3-S1-A732

**Published:** 2015-10-01

**Authors:** J Palamidessi Domínguez, M Valdivia de la Fuente, JJ Rubio Muñoz, S Alcántara Carmona, D Palacios Castañeda, N Martínez Sanz, B Lobo Valbuena, R Fernández Rivas, A Pérez Lucendo, L Pérez Pérez

**Affiliations:** Hospital Puerta de Hierro, Madrid, Spain

## Introduction

Emerging pathogens like Coronavirus (MERS-CoV) and Ebola virus (EV) represent a new challenge for medical personnel and hospital care because of the very specific management that they require.

## Objective

The objective of this study is to evaluate the degree of satisfaction and usefulness of two training courses, based on clinical simulation, focused on the care of patients with MERS-CoV and EV.

## Methods

Prospective study. We evaluated the clinical training courses (October 2014 - January 2015) based on high-fidelity simulation of care in patients with suspected MERS-CoV or EV, carried out in a Clinical Simulation Unit of a tertiary hospital. Both courses were organized in five stations:

1. Course presentation with audiovisual media;

2. Supervised dress-up with the specific personal protective equipment (PPE);

3. High-fidelity simulation in a hospitalization room that was adapted in each of the courses for the specific care of MERS-CoV or EV patients;

4. Supervised withdrawal of PPE and

5. Simulation debriefing.

Degree of resemblance with a real-life situation and degree of academic performance together with student satisfaction were analyzed.

## Results

A total of 288 students, belonging to different health categories and departments, attended 12 courses (6 for MERS-CoV and 6 for EV) during the four month period studied. One hundred and seventy-nine students (10 Intensive Care, 11 Internal Medicine and 11 Pneumology specialists, 77 ICU and Pneumology nurses, 33 ICU and Pneumology nurses assistants and 37 radiology assistants) attended the MERS-CoV course, and 223 (53 physicians, 123 nurses and 47 nurses assistants of the Emergency Department) attended the EV course.

Ninety-four point four percent of the students questioned in the MERS-CoV group and 81,7% in the Ebola group considered that the scenarios played resembled a real-life situation; 82,1% and 88,9%, respectively, acquired skills for the care of these patients; 97,2% and 95,4% described the experience as constructive, and 85,5% and 95,4% would recommend these courses to other professionals.

## Conclusion

Training courses for the care of patients with MERS-CoV and EV infections, based on clinical simulation, resembled real-life situations, achieved a high degree of acceptance, and were considered useful by health professionals, improving the sense of personal safety and quality of care towards these group of potential patients.

